# Correlation between anthropometric measurements and activity level on length and diameter of semitendinosus tendon autograft in knee ligament surgery: A prospective observational study

**DOI:** 10.1051/sicotj/2020007

**Published:** 2020-06-25

**Authors:** Tarun Goyal, Souvik Paul, Lakshmana Das, Arghya Kundu Choudhury

**Affiliations:** 1 Additional Professor, Department of Orthopaedics, All India Institute of Medical Sciences Rishikesh 248201 India; 2 MCh Fellow (Arthroplasty and Joint Reconstruction), Department of Orthopaedics, All India Institute of Medical Sciences Rishikesh 248201 India; 3 Junior Resident, Department of Orthopaedics, All India Institute of Medical Sciences Rishikesh 248201 India

**Keywords:** Anterior cruciate ligament, graft, hamstring, semitendinosus, ligament

## Abstract

*Introduction*: Preoperative estimation of graft parameters can be useful while using hamstring grafts in knee ligament surgeries. Anthropometric parameters may be an easy way to predict the length and diameter of hamstring tendons. A prospective study was conducted to find the correlation between different anthropometric parameters and activity level of the patient on the length and diameter of the graft. Separate regression equations for males and females were also derived for easy prediction. *Methods*: Data were obtained from 95 patients who underwent arthroscopic anterior cruciate ligament reconstruction with autologous hamstring tendon graft. Variables studied were age, sex, height, weight, body mass index (BMI), thigh circumference, thigh length, Tegner activity level, diameter (double and quadruple) and length of semitendinosus tendon graft. *Results*: Height of the patient had strong correlation with graft length (*r* = 0.41, *p* < 0.001), double diameter (*r* = 0.29, *p* = 0.008) and quadruple diameter (*r* = 0.3, *p* = 0.006). Weight of the patients had strong positive correlation with graft length (*r* = 0.34, *p* = 0.002) and quadruple diameter (*r* = 0.34, *p* = 0.002). Thigh length was found to be positively correlating with graft length (*r* = 0.43, *p* < 0.001), double diameter (*r* = 0.29, *p* = 0.007) and quadruple diameter of graft (*r* = 0.34, *p* = 0.002). BMI and thigh circumference of the patients were not found to correlate with graft size. Male patients were found to have longer semitendinosus graft and larger double and quadruple diameter of the graft. There was no association between the Tegner activity scale and graft size. Regression equations between graft length and quadruple diameter and the anthropometric parameters are also derived. *Conclusion*: Height, weight and thigh length are useful anthropometric parameters in the prediction of hamstring tendon size. However, the patient’s Tegner activity level was not found to be associated with size of the hamstring tendon.

## Introduction

Multiple graft choices are available for reconstruction of anterior cruciate ligament (ACL) [1, 2]. Hamstring tendons are one of the most commonly used autografts for reconstruction because of good strength, ease of harvest and lower donor site morbidity. Whereas a predetermined size of graft can be obtained from bone-patellar-tendon, quadriceps tendon and allografts, size of graft made from the hamstring tendon will depend upon the size of tendons harvested during surgery. Adequate size ACL graft is crucial for providing sufficient strength and reducing graft failure [3–5]. Thus, it is important to have an estimate of graft length and diameter preoperatively, so that an alternative graft option may be chosen if the expected size of the graft is not adequate. Use of common anthropometric variables may be an easy estimate of graft size if a good correlation exists between them. Several authors have studied the relationship between graft diameter and length and height, weight, body mass index (BMI) and thigh length of the patients [1, 2, 5–7]. But, none of these studies have studied the relation between the activity level of the individual and graft size. This study prospectively highlights the association of anthropometric parameters (such as age, sex, height, weight, BMI, thigh length and thigh circumference) and activity level of the patients (Tegner activity level) with length and diameter of semitendinosus tendon graft.

## Materials and methods

A total of 95 patients (34 females and 61 males) undergoing arthroscopic ACL reconstruction with ipsilateral autologous hamstring grafts, between January 2017 to October 2018 were included in the study. Patients with medial side knee injury or previous fractures around the knee were excluded. Patients with atrophy of contra-lateral thigh muscles due to any other pathology were also excluded. All patients were operated by the same surgeon. Preoperatively, variables studied were age, sex, height, weight, BMI, thigh circumference (in the bulkiest portion of contralateral thigh approximately 15 cm above the superior pole of the patella), thigh length (from anterior superior iliac spine to medial joint line), and Tegner activity level before the injury. Intra-operatively, semitendinosus graft was harvested through a standard longitudinal incision over pes anserinus after removing all of its extensions to gastrocnemius. The muscle tissue was then removed to skeletonise the tendon. Length of the tendon was recorded using scale over the graft preparation board between the point of its insertion and a point proximally where it starts to taper. The periosteum of anteromedial tibia harvested along with graft was not taken into account while recording graft length. The diameter of the double and quadruple looped tendon was measured using graft sizing block (Arthrex, 4.5–12 mm holes with 0.5-mm increments), considering the smallest diameter hole the graft could pass through.

### Statistical analysis

Statistical analysis was done using SPSS 24. BMI was calculated using the standard formula using height and weight. Descriptive statistics were obtained for all variables. Correlation between variables was studied using Spearman correlation. One-way ANOVA was used to study the association between sex, Tegner activity level and size of graft.

## Results

Data were obtained from 95 patients with a mean age of 30.2 years (18–53, standard deviation of 8.79). Demographic and anthropometric parameters are summarised in Table 1. Male patients were found to have longer semitendinosus grafts and larger double and quadruple diameter of the graft (Table 2).

**Table 1:** Demographic and anthropometric details of patients.

Parameters	Mean	Minimum	Maximum	Standard deviation
Age (years)	30.2	18	53	8.7
Height (cm)	168.1	144	182	7.3
Weight (kg)	72.2	47	94	11.2
Body mass index	25.6	17.3	36.3	3.7
Thigh length	46.9	37	65	4.1
Thigh circumference	47.5	36	62	5.9
Semitendinosus graft length	27.7	20	33	2.6
Doubled graft diameter	6.1	4.5	7.5	0.6
Quadrupled graft diameter	8.4	7	10	0.7

**Table 2:** Graft parameters in mm.

	Mean graft length	Mean graft double diameter	Mean graft quadruple diameter
Male	27.8 ± 2.6 (20–33)	6.1 ± 0.6 (4.5–7.5)	8.5 ± 0.7 (7–10)
Female	27.1 ± 2.6 (21–30)	5.8 ± 0.56 (5–6.5)	7.9 ± 0.6 (7–9)
*p* value	0.39	0.03	0.02

**Figure 1:**
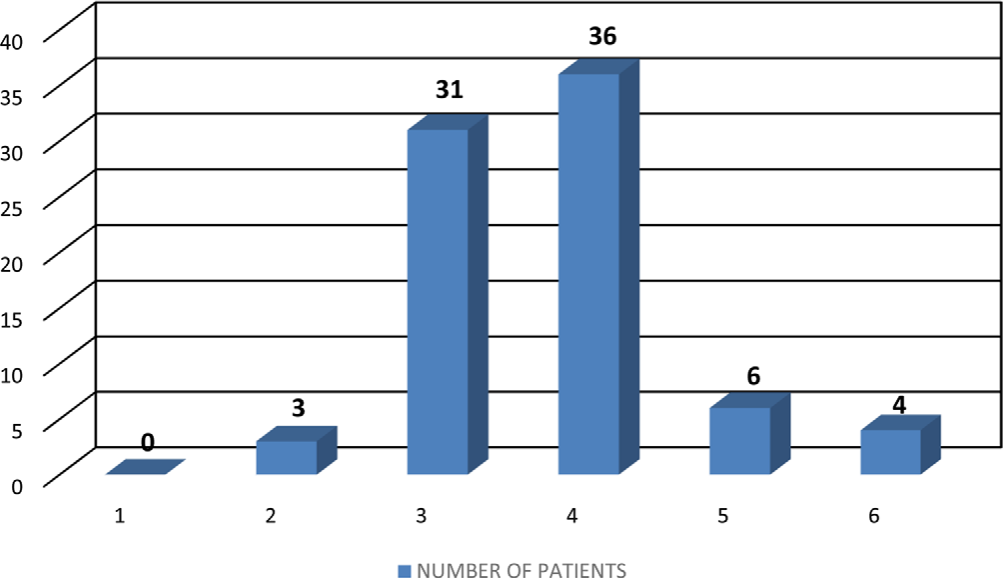
Distribution of Tegner activity scale.

Maximum patients (45%) had a Tegner activity level of 4 (Figure 1). There was no correlation between Tegner activity level and graft length (*r* = −0.06, *p* > 0.05, *n* = 74), double diameter (*r* = −0.07, *p* > 0.05, *n* = 74) and quadruple diameter (*r* = 0.05, *p* > 0.05, *n* = 74).

Correlation between graft size and height, weight and thigh length is summarised in Table 3. BMI and thigh circumference of the patients were not found to have correlation with any of the graft parameters. There was a positive correlation between the age of the patient and graft length (*r* = 0.32, *p* = 0.004, *n* = 95).

**Table 3:** Correlation between graft parameters and height, weight and thigh length.

	Pearson correlation coefficient		
	Graft length	Double diameter	Quadruple diameter
Height	*r* = 0.41, *p* < 0.001	*r* = 0.29, *p* = 0.008	*r* = 0.30, *p* = 0.006
Weight	*r* = 0.34, *p* = 0.002	*r* = 0.2, *p* = 0.06	*r* = 0.34, *p* = 0.002
Thigh length	*r* = 0.44, *p* < 0.001	*r* = 0.29, *p* = 0.007	*r* = 0.34, *p* = 0.002

Simple regression analysis revealed the following equations:

$$\mathrm{Tendon}\;\mathrm{length}\;\left(L\right)\;=\;0.212\times \;\mathrm{thigh}\;\mathrm{length}\;\left(\mathrm{TL}\right)\left(\mathrm{cm}\right)\;+\;0.107\times \;\mathrm{height}\;\left(H\right)\left(\mathrm{cm}\right)-\;0.364\;\left(R^{2}:\;0.118,\;p:\;0.002\right),$$

$$\mathrm{Quadruple}\;\mathrm{diameter}\;\left(\mathrm{QD}\right)\;=\;0.042\times \;\mathrm{thigh}\;\mathrm{length}\;\left(\mathrm{TL}\right)\;+\;0.015\;\times \mathrm{weight}\;\left(W\right)+5.338\;\left(R^{2}:\;0.168,\;p:\;0.000\right).$$

Graft parameters of male patients and female patients were analysed separately. The correlation has been tabulated in Table 4.

**Table 4:** Correlation between graft parameters and height, weight and thigh length in males and females.

	Pearson correlation coefficient					
	Graft length		Double diameter		Quadruple diameter	
	Male	Female	Male	Female	Male	Female
Height	*r* = 0.32, *p* = 0.011	*r* = 0.75, *p* < 0.001	*r* = 0.16, *p* = 0.23	*r* = 0.56, *p* = 0.001	*r* = 0.1, *p* = 0.46	*r* = 0.54, *p* = 0.001
Weight	*r* = 0.26, *p* = 0.04	*r* = 0.7, *p* ≤ 0.001	*r* = 0.14, *p* = 0.27	*r* = 0.32, *p* = 0.06	*r* = 0.26, *p* = 0.04	*r* = 0.46, *p* = 0.007
Thigh length	*r* = 0.4, *p* = 0.001	*r* = 0.6, *p* ≤ 0.001	*r* = 0.29, *p* = 0.02	*r* = 0.24, *p* = 0.16	*r* = 0.32, *p* = 0.01	*r* = 0.33, *p* = 0.05

Simple regression equations for male and female patients separately are as follows:

For male patients:

$$\mathrm{Tendon}\;\mathrm{length}\;\left(L\right)\;=\;0.226\times \;\mathrm{thigh}\;\mathrm{length}\;\left(\mathrm{TL}\right)\left(\mathrm{cm}\right)\;+\;0.111\times \;\mathrm{height}\;\left(H\right)\left(\mathrm{cm}\right)–\;1.75,$$

$$\mathrm{Quadruple}\;\mathrm{diameter}\;\left(\mathrm{QD}\right)\;=\;0.042\times \mathrm{thigh}\;\mathrm{length}\;\left(\mathrm{TL}\right)+0.01\;\times \mathrm{weight}\;\left(W\right)+5.822.$$

For female patients:

$$\mathrm{Tendon}\;\mathrm{length}\;\left(L\right)=0.155\times \;\mathrm{height}\;\left(H\right)\left(\mathrm{cm}\right)+0.094\times \mathrm{weight}\;\left(W\right)\left(\mathrm{kg}\right)-4.536,$$

$$\mathrm{Quadruple}\;\mathrm{diameter}\;\left(\mathrm{QD}\right)=0.039\times \mathrm{height}\;\left(H\right)\left(\mathrm{cm}\right)+1.585.$$

## Discussion

**Figure 2:**
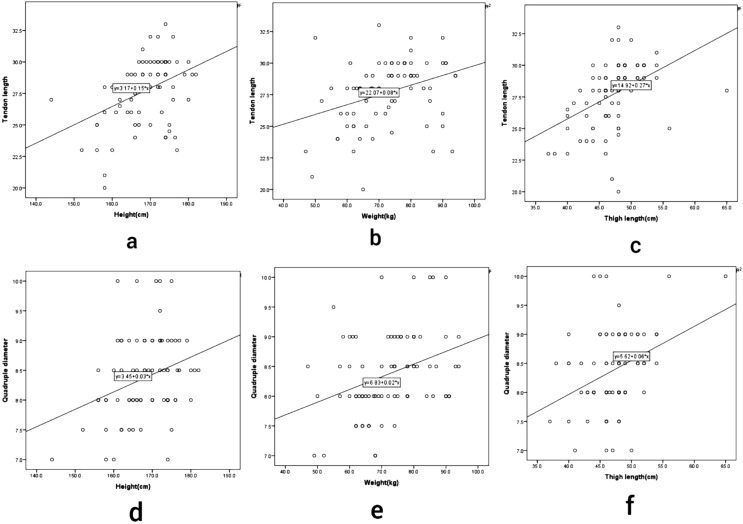
Scatter plots showing correlations of tendon length to height (a), weight (b) and thigh length (c) and also correlations of quadruple diameter to height (d), weight (e) and thigh length (f).

Hamstring tendon grafts are one of the most popular autograft choices in ACL reconstruction. But their length and diameter may be unpredictable. Biomechanical studies have shown increased strength and stiffness of a hamstring graft with increased graft diameter [3, 4]. Patients having graft diameter of more than 8 mm have been shown to have lesser revision rates and graft failures [8, 9]. Female patients were found to have lesser graft lengths and diameters compared to male patients (Table 2). This finding has been reported by several authors previously. Need for estimating graft length and diameter preoperatively may be more important in female patients. Height is the most predictable indicator of graft diameter and length in the literature [2, 5–7, 10–12]. This study showed a strong correlation between height and graft length (*r* = 0.41, *p* < 0.001), double diameter (*r* = 0.29, *p* = 0.008) and quadruple diameter (*r* = 0.3, *p* < 0.001). A statistically significant correlation between weight and graft length (*r* = 0.34, *p* = 0.002) and quadruple diameter (*r* = 0.34, *p* = 0.002) was also noted, which has been reported in only a few studies [2, 7, 11]. BMI did not show any correlation with the graft parameters. Leg length has been found to have a role in predicting the graft parameters in the literature [12, 13]. Thigh-length was taken as an anthropometric parameter in this study, which has been rarely described in previous studies [14, 15]. It was found to be significantly correlating with graft length (*r* = 0.44, *p* < 0.001), double (*r* = 0.29, *p* = 0.007) and quadruple diameter (*r* = 0.35, *p* = 0.002) (Figure 2). Thus, thigh-length might be easy to measure and sensitive tool for the prediction of graft length and diameter. Naiyer et al. [6] had shown thigh circumference as a predictive parameter, but their result has not been replicated in this study or any other previous studies [7, 11, 16].

Correlation between Tegner activity scale and graft parameters was also evaluated, which has not been studied before. It is plausible that patients with higher activity levels may have larger tendon sizes. No significant correlation was found between the two.

The study also highlights easy and reliable regression equations for pre-operatively determining the tendon lengths and quadruple diameter from these data.

$$\begin{array}{c} L\;=0.212x\;\mathrm{TL}\;\left(\mathrm{cm}\right)+0.107x\;H\;\left(\mathrm{cm}\right)=0.364,\;\\ \mathrm{QD}=0.042x\;\mathrm{TL}+\;0.015x\;W+5.338. \end{array}$$

Separate simple regression equations for both male and female patients could also be formulated as stated below:

$$\begin{array}{c} \mathrm{For}\;\mathrm{male}\;\mathrm{patients}:\;L\;=\;0.226\;\times \;\mathrm{TL}\;\left(\mathrm{cm}\right)+0.111\;\times \;H\;\left(\mathrm{cm}\right)\;-1.75,\;\mathrm{QD}=\;0.042\;\times \;\mathrm{TL}+0.01\;\times \;W+5.822;\\ \mathrm{For}\;\mathrm{female}\;\mathrm{patients}:\;L\;=\;0.155\;\times \;H\;\left(\mathrm{cm}\right)+0.094\;\times \;W\;\left(\mathrm{kg}\right)-4.536,\;\mathrm{QD}=\;0.039\;\times \;H\;\left(\mathrm{cm}\right)+1.415. \end{array}$$

The findings from this study suggest that to achieve a quadruple-diameter of 8 mm with isolated semitendinosus tendon, the patient should have 155 cm height, 58 kg weight and 40 cm thigh length. Thus considering gender-specific equations, a female patient should have 168 cm height and a male patient should have 60 kg weight and 38 cm thigh length to obtain a quadruple graft diameter of 8 mm. According to the dataset, thigh-length has proven to be a more useful predictor in case of males, whereas height was correlating more significantly in females. This can help to avoid the intraoperative complication of having a small hamstring tendon. The patient can be counseled well before surgery about the need for any other tendon as a graft option. This study is important because data on the correlation of anthropometric parameters from Indian population are limited [6, 12], and no previous studies have studied the effect of activity level of the patient on graft parameters and sexual differences in graft sizes. Finally, the data were unique as all the grafts were harvested by the same surgeon and following the same technique without any chances of bias.

## Conclusion

The ability to predict the graft parameters preoperatively is undeniably useful. Common anthropometric parameters like height have been proven useful in several studies. However, this study found the weight of the patient and thigh length also as good predicting parameters for quadruple diameter and length of the graft. Pre-injury Tegner activity level fails to prove a useful parameter. Male patients with height lesser than 155 cm, weight lesser than 58 kg and thigh length lesser than 40 cm are not only at risk of a thinner graft, but also the tendon length may be shorter needing alternative graft options.

## Conflict of interest

The author(s) declared no potential conflicts of interest with respect to the research, authorship, and/or publication of this article.
